# Comparative Phytonutrient Analysis of Broccoli By-Products: The Potentials for Broccoli By-Product Utilization

**DOI:** 10.3390/molecules23040900

**Published:** 2018-04-13

**Authors:** Mengpei Liu, Lihua Zhang, Suk Lan Ser, Jonathan R. Cumming, Kang-Mo Ku

**Affiliations:** 1 School of Food and Bioengineering, Zhengzhou University of Light Industry, Zhengzhou 450002, China; mengpei0402@gmail.com (M.L.); zhanglihua82828@163.com (L.Z.); 2 Collaborative Innovation Center for Food Production and Safety, Zhengzhou University of Light Industry, Zhengzhou 450002, China; 3 Department of Biochemistry, West Virginia University, Morgantown, WV 26506, USA; suser@mix.wvu.edu; 4 Department of Biology, West Virginia University, Morgantown, WV 26506, USA; jcumming@wvu.edu; 5 Division of Plant and Soil Sciences, West Virginia University, Morgantown, WV 26506, USA

**Keywords:** broccoli, by-products, glucosinolates, carotenoid, chlorophyll, mineral, vitamins E and K, phenolic compounds, antioxidant activity

## Abstract

The phytonutrient concentrations of broccoli (*Brassica oleracea* var. italica) florets, stems, and leaves were compared to evaluate the value of stem and leaf by-products as a source of valuable nutrients. Primary metabolites, including amino acids, organic acids, and sugars, as well as glucosinolates, carotenoids, chlorophylls, vitamins E and K, essential mineral elements, total phenolic content, antioxidant activity, and expression of glucosinolate biosynthesis and hydrolysis genes were quantified from the different broccoli tissues. Broccoli florets had higher concentrations of amino acids, glucoraphanin, and neoglucobrassicin compared to other tissues, whereas leaves were higher in carotenoids, chlorophylls, vitamins E and K, total phenolic content, and antioxidant activity. Leaves were also good sources of calcium and manganese compared to other tissues. Stems had the lowest nitrile formation from glucosinolate. Each tissue exhibited specific core gene expression profiles supporting glucosinolate metabolism, with different gene homologs expressed in florets, stems, and leaves, which suggests that tissue-specific pathways function to support primary and secondary metabolic pathways in broccoli. This comprehensive nutrient and bioactive compound profile represents a useful resource for the evaluation of broccoli by-product utilization in the human diet, and as feedstocks for bioactive compounds for industry.

## 1. Introduction

**Figure 1:**
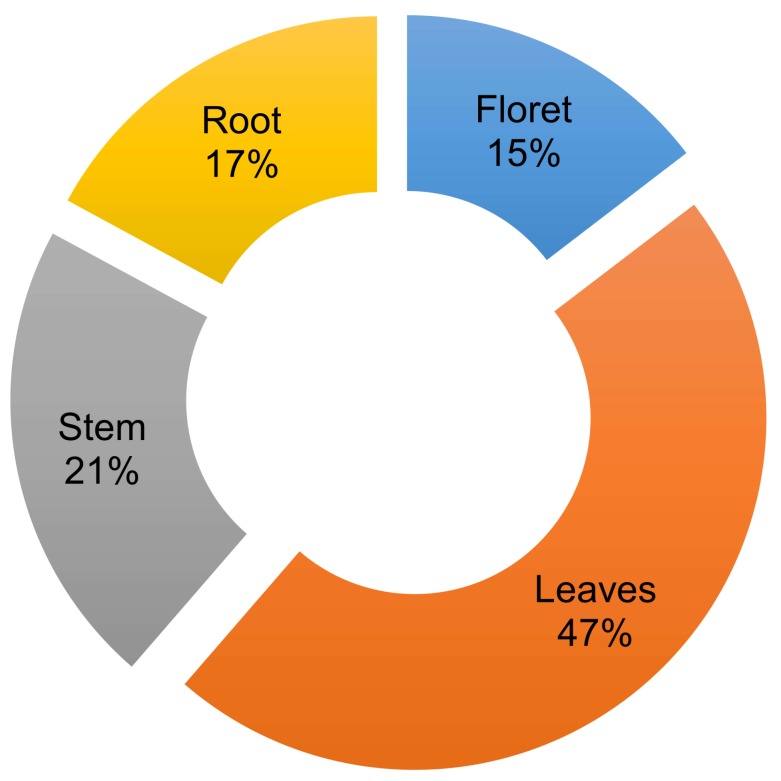
Broccoli (“Gypsy” cultivar, grown at the West Virginia Agronomy farm 2016 with conventional practice) individual tissue biomass (fresh weight) percentage to total biomass. The data collected from seven individual mature broccoli plants. Average of total biomass was 776 g per plant.

Humans have domesticated brassica crops to utilize specific tissues for human consumption [1]. A significant portion of some brassica crops are not harvested or utilized, however, which is a critical issue in current agricultural production systems. One such example is broccoli, from which only ~10–15% of the total aerial biomass of the plant is consumed (Figure 1) [2]. Our previous study showed that broccoli root contains high amounts of glucosinolate and quinone reductase detoxifying enzyme-inducing activity [3]. However, utilizing broccoli root tissue is not easy because of the difficulty in harvesting and processing the lignified root tissues. Although broccoli and collard greens belong to the same species (*Brassica oleracea*), and collard leaf is utilized as the edible part, broccoli leaf is seldom utilized for food. Some people consume broccoli stems, but this is not the norm. Commercial broccoli florets usually have ~10 cm of attached stem. Although the stems nearest to florets are tender and edible, the bottom stem is lignified and not acceptable for food consumption. The potential consumption of stems and leaves would increase productivity and sustainability of the World’s broccoli crop by increasing yield from 15% up to as much as 83% (Supplementary Material Figure S1).

Broccoli production in the United States in 2015 was 17.6 million tons [3]. If more of the aerial part of the broccoli plant can be processed for consumption, the waste amount from production would be significantly reduced, realizing additional profit to farmers. Since broccoli leaves and stems are seldom utilized, information on the nutritional benefits and human health-promoting compounds in them is rare, although antioxidant activity, in vitro anticancer growth bioactivity, and the effect of broccoli by-product feeding on lactating performance of dairy cows and meat quality of broiler chickens have been previously reported [4,5,6,7,8]. To stimulate the utilization of broccoli leaf and stem tissues, it is essential to evaluate their human nutritional values.

Sulforaphane is a well-known anticancer compound present in broccoli [3]. Glucoraphanin is the precursor of sulforaphane, but the amount of sulforaphane conversion depends on myrosinase binding enzymes (epithiospecifier protein, ESP; epithiospecifier modifier 1, ESM1) and other factors [9,10,11]. Thus, the sulforaphane conversion can be significantly changed by genetic and environmental factors and represents an opportunity for nutritional modification of broccoli by-products. Chlorophylls, the green pigments capturing solar energy for photosynthetic reactions, have antimutagenic, antioxidant, antiviral, and anticancer activities [12,13,14,15]. Carotenoids, plant pigments that augment light energy capture, also have potential health benefits against cancer and cardiovascular and photosensitivity disorders [16,17]. Mineral nutrients are essential for many human physiological functions and vitamins, such as vitamin E and K, are oil soluble, and also have essential human health benefits [18,19]. Phenolic compounds found in plant tissues have antioxidant, hepatoprotective, renal function protective, analgesic, and anti-inflammatory effects [20,21,22]. Thus, the potential nutritive benefits of broccoli by-products represent an opportunity to augment human diets. Recently, “BroccoLeaf™” (Salinas, CA, USA) has come to market (http://thebroccoleaf.com/). The United States Department of Agriculture (USDA) National Nutrient Database has comprehensive nutrient data [23] for broccoli florets, leaves, and stalks. However, some important nutrients (vitamin E and K, carotenoids, and glucosinolates) of broccoli stalks and leaves are lacking. Therefore, the objective of this study was to compare the nutritional values among different broccoli tissues, including floret, stem, and leaf. 

## 2. Results and Discussion

### 2.1. Broccoli Primary Metabolites

**Figure 2:**
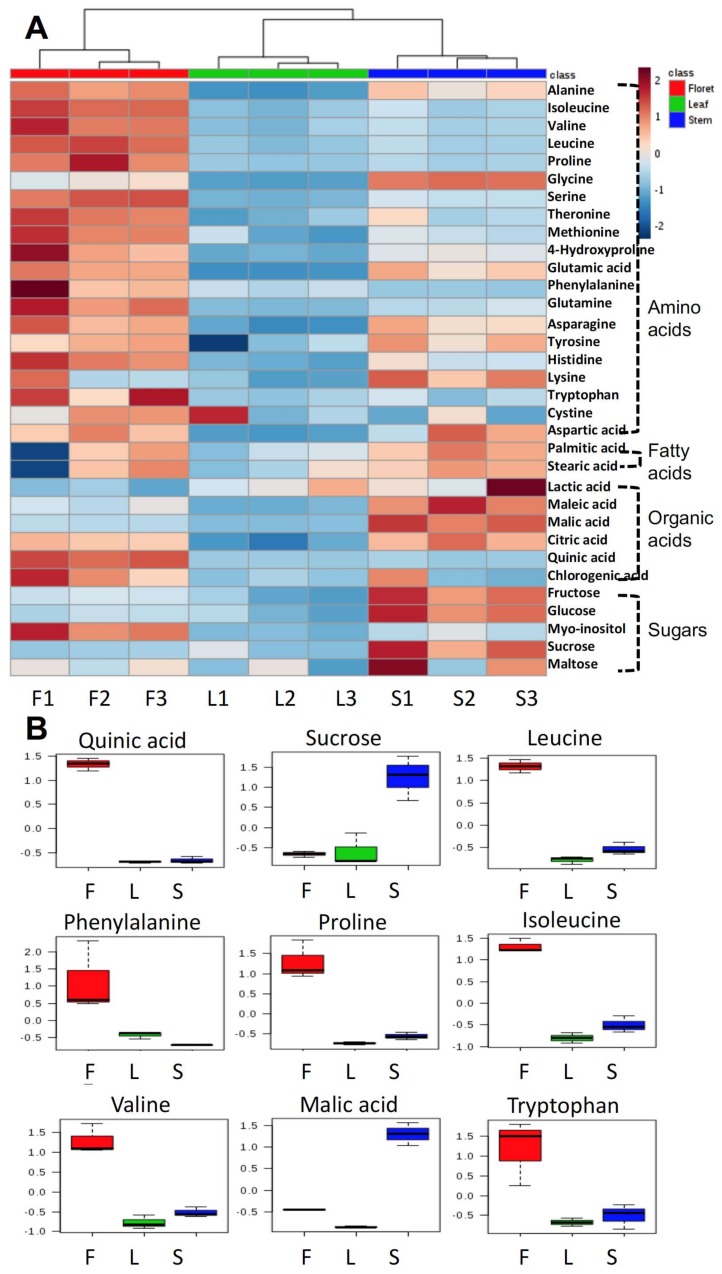
Primary metabolites in different tissues of broccoli (amino acids, fatty acids, organic acids, and sugars) in heatmap (**A**) and the top nine selected biomarkers based on variable importance in projections (**B**); F, L, and S indicate floret, leaf, and stem, respectively. The number after each tissue code (F, L, or S) indicates biological replication.

The primary metabolite profile of broccoli floret tissue was significantly different from that of stem or leaf (Figure 2A). The concentrations of most amino acids in floret tissue were higher than those in the other tissues. In contrast, most sugars (fructose, glucose, sucrose, and maltose) were higher in stems than other tissues. The top nine biomarkers are presented in Figure 2B based on variable important projection (VIP) values. Quinic acid, sucrose, leucine, phenylalanine, proline, isoleucine, valine, malic acid, tryptophan were the most important discriminatory biomarkers. Except for sucrose and malic acid, these biomarkers were significantly higher in floret tissue than the other tissues (Figure 2B).

### 2.2. Glucosinolates

Eleven glucosinolates (6 aliphatic, 1 aromatic, and 4 indole) were detected in various broccoli tissues (Table 1). The total glucosinolate concentration of floret (34.66 μmol/g dry weigh, DW) tissue was significantly higher than that in stem (7.45 μmol/g DW) and leaf (10.08 μmol/g DW) tissues. The total aliphatic glucosinolate concentration (glucoiberin, progoitrin, glucoraphanin, sinigrin, glucoerucin, and gluconapin) in floret tissue (14.95 μmol/g DW) was significantly higher than in stem (5.99 μmol/g DW) and leaf (3.52 μmol/g DW) tissues. All individual glucosinolate concentrations in floret tissue were higher than stem and leaf tissues except for glucoerucin. Interestingly, the glucoerucin concentration of stem (0.89 μmol/g DW) was about 10- and 20-fold higher than floret and leaf tissues, respectively. The total indole glucosinolate concentration (glucobrassicin, neoglucobrassicin, 4-hydroxyglucobrassicin, and 4-methoxyglucobrassicin) was also higher in broccoli florets (18.82 μmol/g DW) than in stems (1.44 μmol/g DW) and leaves (6.45 μmol/g DW). Notably, the concentration of neoglucobrassicin in floret tissue was 16.67 μmol/g DW, which was significantly higher than the other two tissues (1.11 μmol/g DW in stems and 5.78 μmol/g DW in leaves). This elevated neoglucobrassicin concentration may be an indicator of insect damage due to the no-pesticide regime used in this study. We previously reported that neoglucobrassicin in broccoli can be increased about five-fold by 250 µM methyl jasmonate treatment, a surrogate for insect-induced damage, compared to control [24,25] and, further, the increased neoglucobrassicin more effectively controls the growth and development of the cabbage looper caterpillar compared to glucobrassicin [26]. The hydrolysis product of neoglucobrassicin, *N*-methoxyindole-3-carbinol, has a weak quinone reductase-inducing activity [25]. Interestingly, it has also been reported that *N*-methoxyindole-3-carbinol more effectively arrests the colon cancer cell cycle than indole-3-carbinol [27]. The major glucosinolates in all broccoli tissues were glucoraphanin and neoglucobrassicin. Although the absolute concentration of glucoraphanin in floret tissues was the highest among the different broccoli tissues, the relative percentage of glucoraphanin to total glucosinolates in florets (32.5%) was similar with leaves (27.5%) but lower than that in stems (48.9%). This result indicates that stems and leaves of broccoli also contain considerable amounts of health-promoting compounds.

### 2.3. Nitrile Formation Percentage to Total Glucosinolate Hydrolysis Products and Myrosinase Activity

**Figure 3:**
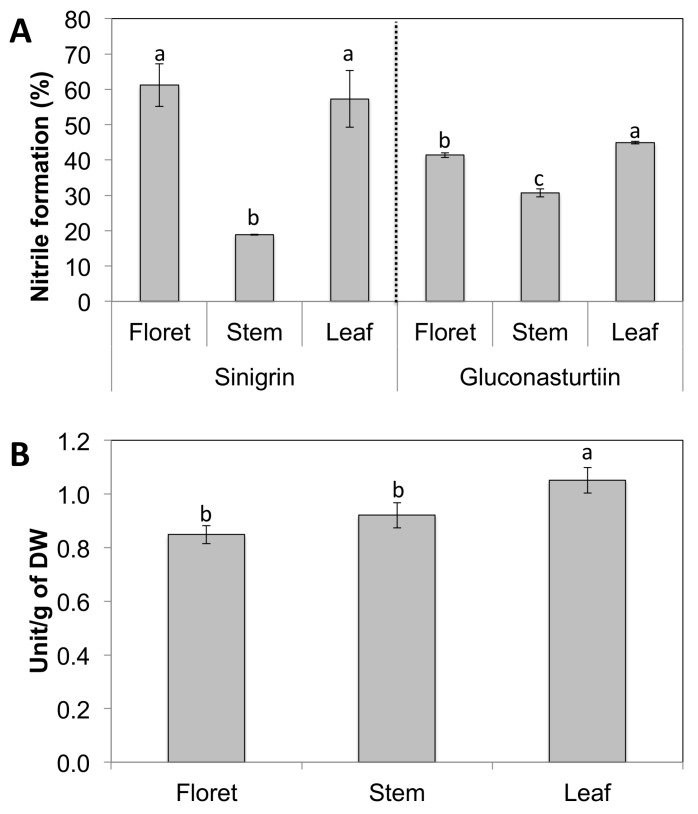
Nitrile formation is shown as the relative ratio of nitrile to the total hydrolysis product formed (sum of isothiocyanates and nitriles). Data are presented as the mean concentration ± standard deviation (*n* = 3). Different letters above the error bar indicate significant differences between different tissues within the same glucosinolate substrate by Student’s *t*-test at *p* ≤ 0.05. One unit was defined as 1 μmol of hydrolysis products of glucosinolates released per min.

Broccoli floret and leaf tissues had significantly higher nitrile formation compared to broccoli stems regardless of substrate used (Figure 3A). Nitrile formation of broccoli florets was 100% higher than stem tissue on the sinigrin substrate, while nitrile formation of broccoli florets was only 33% higher than stem tissue on gluconasturtiin. This difference in nitrile formation may be due to the tissue-specific myrosinase expression and/or levels of the myrosinase cofactors ESP and ESM1 that regulate its activity. Another possibility is that different tissue-specific myrosinases in floret and stem tissues have different affinity for different glucosinolate substrates. Broccoli leaf tissue had the highest myrosinase activity between broccoli tissues, and leaf and stem had statistically identical levels of myrosinase activity (Figure 3B). The regulation of the pathways metabolizing glucosinolate is important in directing the health benefits of broccoli tissues, because products of their hydrolysis by myrosinase generate sulforaphane, phenethyl isothiocyanate, and allyl isothiocyanate, potent anticancer compounds [28,29]. 

### 2.4. Gene Expression of Glucosinolate Biosynthesis and Myrosinase-Related Genes

**Figure 4:**
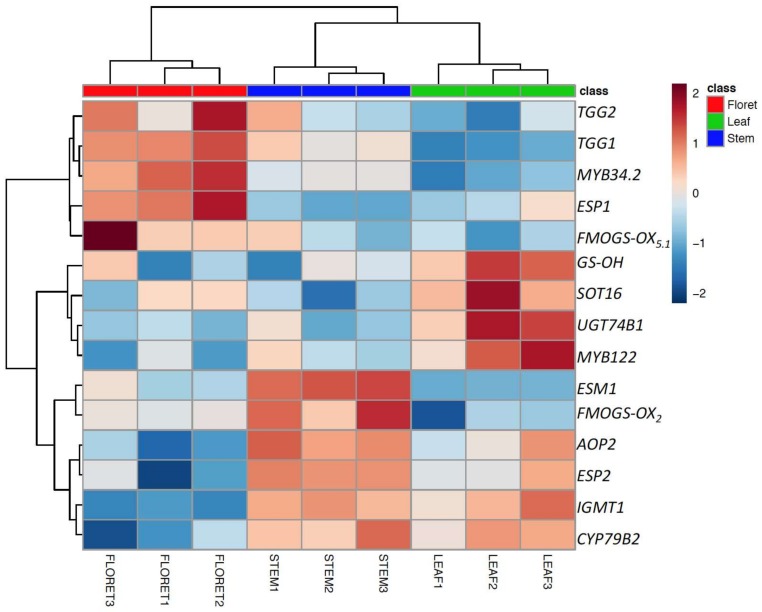
Top 15 significantly expressed genes (glucosinolate biosynthesis, myrosinase, cofactors genes) among the different broccoli tissues. The gene expression values were normalized based on mean value of floret and then transformed to logarithmic scale.

Among 17 target genes, 15 significantly differentially-expressed genes are presented as a heatmap in Figure 4. Although myrosinase activity of broccoli leaf was the highest among broccoli tissues (Figure 3B), the expression of the two myrosinase genes, *TGG1* and *TGG2*, and the transcription factor *ESP1* were significantly higher in floret tissue compared to leaves, as well as stems (Figure 4). Thus, myrosinase activity could not be explained by the expression levels of *TGG1* and *TGG2* alone, suggesting potentially tissue-specific myrosinase homologs [28]. We found that two *ESP* gene homologs were differentially expressed in specific tissues; for example, *ESP* was highly expressed in floret tissue, whereas *ESP2* was highly expressed in leaf tissue, which is consistent result with our previous findings [3]. The gene expression levels of *ESP2* and *ESM1* were significantly higher in stem tissue (Figure 4). The lower expression of *ESP1* (stem has 50-fold lower than floret) and higher expression of *ESM1* (stem has 6-fold higher than floret) support the observed lower nitrile formation in stems compared to the other tissues. Controlling ESP enzyme activity has been suggested as an effective way to convert glucoraphanin to sulforaphane [29]. However, the current results and findings from previous work [3] suggest that controlling *ESM1* may also be an effective way to produce more isothiocyanate. The low gene expression levels for indole glucosinolate transcription factors (*MYB34.2* and *MYB122*) and glucosinolate core structure synthesis genes (*UGT74B1* and *SOT16*) supports low accumulation of indole glucosinolates in broccoli stem tissue. The gene expression level of *AOP2* in floret tissue was lower than leaf tissue, consistent with previously reported glucoraphanin accumulation in broccoli floret tissue [3].

### 2.5. Carotenoids and Chlorophylls

β-Carotene, violaxanthin, neoxanthin, and lutein were detected in the different tissues of broccoli (Table 2). Stem tissues did not have β-carotene and violaxanthin, whereas leaf tissue had the highest concentrations of β-carotene, violaxanthin, neoxanthin, and lutein (248, 206, 156, and 484 µg/g DW, respectively). Regardless of tissue type, lutein accounted for the largest proportion of the total carotenoid pool, representing 47.2, 69.2, and 44.2% in floret, stem, and leaf, respectively. Leaf tissue had higher total carotenoid concentration (1095 µg/g DW) than florets (181 µg/g DW) and stems (15.6 µg/g DW).

Some carotenoids, such as α-carotene and β-carotene, possess provitamin A activity, which is beneficial for eye health [30]. Leaf tissue had the highest recommended daily allowance (RDA) of vitamin A, which accounted for 32.0% and 42.9% for males and females (>14 years), respectively. In contrast, floret alone provided 3.0% and 3.9% of RDA for males and females [31]. Thus, broccoli leaf tissue can be an excellent vitamin A source compared to broccoli floret. 

The concentrations of chlorophyll *a* in florets, stems, and leaves were 852, 144, and 4478 µg/g DW, whereas the concentration of chlorophyll *b* was 135, 23, and 781 µg/g DW, respectively (Table 2). In this study, the broccoli leaf and floret tissues contained about 5–7 times of chlorophyll *a* than of chlorophyll *b*, which is higher than the previous data evaluated with four broccoli varieties [32]. The different result may reflect the ratio of broccoli flower buds and stems included in sampling protocols, as well as differences in environmental conditions between the two studies [33,34]. 

### 2.6. Essential Mineral Nutrients

Nine minerals were analyzed from the different broccoli tissues (Table 3). Floret tissue was the highest in Fe, Zn, and P, whereas leaf tissue had the highest Mn and Ca, and the stem had the highest Na. There were no significant differences in concentrations of Cu, Mg, or K among tissues. Leaves contained 6-fold higher Ca and 1.4-fold higher Mn concentrations than florets, which may reflect the delivery of Ca and Mn to leaves in the transpiration stream in the xylem versus to the florets via the phloem. Calcium is phloem immobile, as is Mn, and the delivery of these to developing florets will be limited in relation to other mineral elements, such as Zn and P [35,36]. The limited Na concentrations in leaves and especially florets point to the existence of effective Na exclusion systems in broccoli, which may contribute to its healthy mineral-element profile.

RDA (%) was calculated for the different tissues of fresh broccoli weight basis (100 g). Among them, the RDA of Mn (22.0%) and Ca (46.6%) in leaves were significantly higher than those for florets. However, florets were the best source of Zn with an average RDA value of 6.8%.

### 2.7. Vitamins E and K

**Figure 5:**
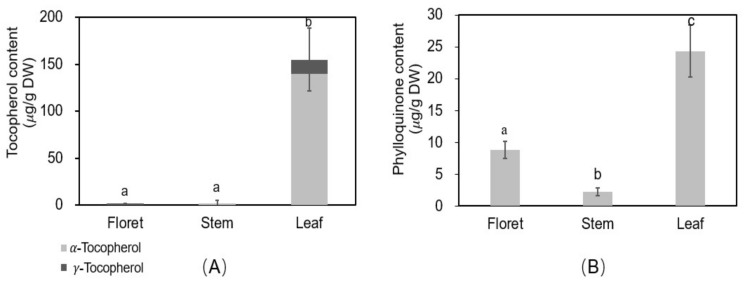
Oil soluble vitamin concentrations in different tissues of broccoli. Tocopherol (vitamin E) (**A**) and phylloquinone (vitamin K1) (**B**); Different letters above the error bars indicate significant differences among the different broccoli tissues by LSD at *p* ≤ 0.05.

Vitamin E consists of four tocopherols and four tocotrienols, of which α- and γ-tocopherols are the predominant compounds in broccoli, red sweet pepper, carrot, and vegetable oils [37,38]. There was a significant difference in tocopherols among different tissues of broccoli (Figure 5A). The concentration of total tocopherols was 1.57, 1.97, and 155 µg/g DW in floret, stem, and leaf, respectively, and α-tocopherol accounted for 0, 100, and 90.4% of total tocopherols of each tissue. Among different broccoli tissues, leaf was the highest in α- and γ-tocopherols. 

The recommended intake of vitamin E is 15 mg/day for adults according to the USDA nutrient database [39]. A 100 g portion of fresh florets, stems, or leaves of broccoli can provide 0.15, 0.14, or 19.9% of the RDA, respectively. Vitamin E has potential benefits against cardiovascular disease, inflammation, diabetes, prostate cancer, and Alzheimer’s disease [18]. Therefore, broccoli leaf could be the excellent source to provide vitamin E for the human diet.

Phylloquinone (vitamin K1) is considered the dominant form of dietary vitamin K (>90%) [40]. The phylloquinone concentrations of floret, stem, and leaf tissues were 8.84, 2.21, and 24.3 µg/g DW, respectively. Leaf tissue had 2.8-fold higher phylloquinone concentrations than floret tissue. The concentration for florets agrees with previous reports (102 µg/100 g fresh weight of phylloquinone [19] vs. 123 µg/100 g fresh weight in the current study). The higher concentrations in leaves noted here have not previously been reported.

The recommended intake of vitamin K for adult women and men is 90 and 120 µg/day, respectively, based on the USDA nutrient database [41]. The leaf and floret tissues can provide 521–391% (adult women/men) and 137–102% (adult women/men) of the RDA, respectively, based on 100 g portions of fresh tissues. However, stem tissue only provides 27% (women) and 20% (men) of the RDA for adults, respectively. Vitamin K has health-beneficial effects on blood coagulation, bone health, and in reducing the risk of vascular calcification and cardiovascular disease [42]. The results of this study showed leaf tissue is an excellent source of phylloquinone. 

### 2.8. Total Phenolic Concentrations and Free Radical Scavenging Activity

**Figure 6:**
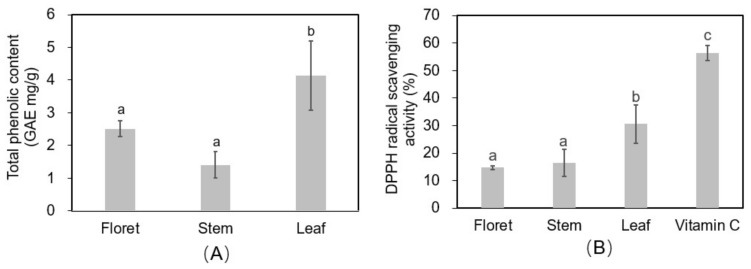
Total phenolic content (gallic acid equivalent (GAE) mg/g DW) (**A**) and 2,2-diphenyl-1-picryl-hydrazyl-hydrate (DPPH) antioxidant activity (**B**) in different tissues of broccoli extract (12.5 mg DW/mL of 70% methanol). Vitamin C was used as positive control (125 µg/mL).

Leaves had 1.6-fold and 2.9-fold higher total phenolic concentrations (4.14 mg gallic acid equivalents/g DW) than florets (2.51 mg GAE/g DW) and stems (1.41 mg GAE/g DW) (Figure 6A). The 2,2-diphenyl-1-picryl-hydrazyl-hydrate (DPPH) assay was performed on extracts from the different broccoli tissues as an estimate of the reactive oxygen species (ROS)-scavenging activity of antioxidants in the tissues (Figure 6B). Vitamin C (ascorbic acid) was used as a positive control at a concentration of 125 µg/mL in 70% methanol, and had a DPPH radical scavenging activity of 56.3%. Broccoli leaf tissue had a significantly higher DPPH radical scavenging activity (30.7%) than stem (16.4%) or floret (14.7%) tissues (Supplementary Material Figure S2). Both the phenolic and ROS scavenging findings are supported by previous reports [5]. Although our findings align in general with those of Hwang et al. [5], these authors also found large variation among cultivars, with “Kyoyoshi” exhibiting 2.43-, 1.49-, and 3.23-fold greater total phenolic compounds in leaves, stems, and florets, respectively, compared to two other broccoli cultivars. This implies there is signficant genetic variation in antioxidant compounds in broccoli tissues, which should be considered by growers or crop breeders for more sustainable and productive broccoli systems, especially in terms of by-product utilization.

### 2.9. Potential of Broccoli By-Products for Human Consumption and Nutraceutical Industries

Food production is limited by resources across much of the planet, and increasing the use of crop by-products represents an opportunity to reduce scarcity worldwide. The industrial manufacturing of fruits and vegetables, for example, generates on average approximately 50% agrowaste by-product [43,44]. According to the Food and Agriculture Organization (FAO), total world production of broccoli (and cauliflower, which has similar by-product challenges) in 2009 was 19,845,519 tons [45]. Given the disproportionately high by-product percentage (~90%) during harvest as well as during processing of this crop [45], large amounts of the broccoli plant are not utilized. Further, the by-product percentage of broccoli may increase substantially under NaCl stress [2], suggesting that inadequate irrigation or sodic soils may produce even more by-product at the expense of edible portions. 

Broccoli by-product generation in the United States in 2015 was estimated at 158 million tons [3], representing a large potential resource for consumption or alternative use. Broccoli by-product has been used traditionally as an animal feedstuff for broiler chickens and cows [7,8,46]. Several investigators have further explored broccoli by-products as antimicrobial agents for foodborne bacteria or soil-borne bacteria [47,48], since the hydrolysis products of glucosinolate have antimicrobial activity. Recent studies have also attempted to incorporate broccoli by-products as additives to improve the quality or nutritional value of various foods [6,49]. These and other recent papers highlight the feasibility of utilization of broccoli by-products for the human diet.

Additionally, broccoli by-products may be used as a feedstock for bioactive compound production [50]. Hwang and Lim [5] reported that broccoli leaf extracts had significantly higher cell growth inhibitory activities on human lung carcinoma (NCI-H1229) and human colon adenocarcinoma (HT-29) cell lines than corresponding floret extracts. They also reported strong positive correlations between total phenolic compounds and cancer cell growth inhibition. Thus, the high concentrations of metabolites in broccoli leaves may be valuable to the nutraceutical or pharmaceutical industries. However, these recent publications did not provide specific nutrient information on broccoli extracts, and our results fill this gap by providing information on vitamin E and K that has been not reported previously, as well as nitrile formation ability from different tissues. Linking the metabolomic profiles, secondary metabolite chemistry, and gene expression patterns underlying glucosinolate production in different broccoli by-products, we have presented fundamental information that may be used by breeders and others to modify broccoli to increase health benefits and provide sources of bioactive compounds for industry [28,29]. Utilization of by-products for the human diet and as new sources of anticancer and anti-inflammatory compounds is an excellent utilization of agrowaste that will not only benefit human health but will provide new income streams for growers as well. Some countries have advanced by-product utilization, including for pepper leaf (*Capsicum annum*) [51,52], pumpkin leaf (*Telfairia occidentalis* Hook f.), sweet potato leaf (*Ipomea batatas*), cassava leaf (*Manihot esculenta* Crantz) [53], and soybean leaf [54]. Might broccoli leaf be next edible leafy vegetable?

## 3. Materials and Methods

### 3.1. Broccoli Production 

**Figure 7:**
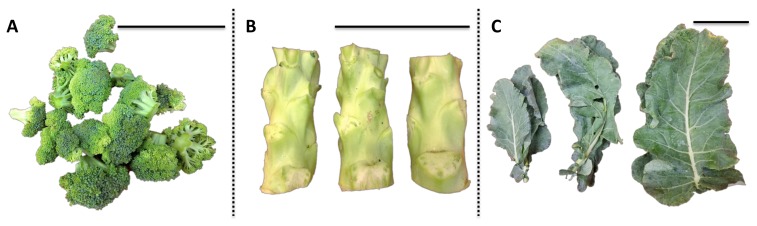
Representative image of various broccoli tissues for one biological replication. The black scale bar at the right upper corner of each picture is 10 cm. (**A**) Broccoli floret; (**B**) broccoli stem; (**C**) broccoli leaf.

“Green Magic” broccoli seeds (Johnny’s Selected Seeds) were sowed on 4 May 2017 and germinated in flats filled with Sunshine #1 Mix (Sun Gro Horticulture, Vancouver, BC, Canada) in the greenhouse facility at the West Virginia University (WVU), and were allowed to grow in the greenhouse for four weeks under a 25/18 °C and 14/10 h day/night temperature/photoperiod regime with supplemental lighting. After four weeks, the broccoli seedlings were transplanted to the WVU Agronomy Farm on 6 June in a randomized complete block design with three replicates. Various broccoli tissues, including florets, stems, and leaves, were harvested on 15 August as bulked samples from three mature plants for one biological replication (*n* = 3). For leaf, one each apical, median internode, and basal mature leaves were collected and pooled for one biological replication. Representative images of each tissue are shown in Figure 7. Water content of each broccoli tissue (floret 86%; stem 89%; leaf 81%) was calculated before and after freeze-drying and used to convert subsequent analyses to fresh weight basis for USDA recommended dietary allowance (RDA) values.

### 3.2. Primary Metabolite Extraction and Analysis

Primary metabolites were extracted following published protocols [55] with modifications of extraction solvent volume. Samples (50 mg) were weighed into 2 mL microcentrifuge tubes, followed by the addition of 80 μL of ribitol (10 mg/mL) as an internal standard, and extracted with 1.4 mL of 70% methanol at 75 °C. After cooling, sample extracts were centrifuged at 15,000*g* for 3 min, and 0.7 mL supernatant samples were transferred to new 2 mL microcentrifuge tubes. To fractionate polar compounds, 0.375 mL of cold chloroform (–20 °C) and 0.7 mL cold water (4 °C) were added. After vigorous mixing, the extracts were centrifuged at 15,000*g* for 3 min, and 50 μL supernatant samples were transferred to 1.5 mL microcentrifuge tubes. The extracts were dried using a Vacufuge concentrator (Eppendorf, Thermo Fisher Scientific, Waltham, MA, USA) with 10 μL of methanol to facilitate water evaporation. Dried extracts were derivatized with 50 μL methoxyamine hydrochloride (40 mg/mL in pyridine) for 90 min at 37 °C, then with 70 μL N-methyl-N-trimethylsilyl-trifluoroacetamide (MSTFA) + 1% trimethylchlorosilane (TMCS) at 37 °C for 30 min. Metabolites were analyzed using a GC-MS (Trace 1310 GC, Thermo Fisher Scientific) coupled to a MS detector system (ISQ QD, Thermo Fisher Scientific) and an autosampler (Triplus RSH, Thermo Fisher Scientific). A capillary column (Rxi-5Sil MS, Restek, Bellefonte, PA, USA; 30 m × 0.25 mm × 0.25 µm capillary column w/10 m Integra-Guard Column) was used to detect polar metabolites. After an initial temperature hold at 80 °C for 2 min, the oven temperature was increased to 330 °C at 15 °C/min and held for 5 min. Injector and detector temperatures were set at 250 °C and 250 °C, respectively. An aliquot of 1 μL was injected with the split ratio of 70:1. The helium carrier gas was kept at a constant flow rate of 1.2 mL/min. The mass spectrometer was operated in positive electron impact mode (EI) at 70.0 eV ionization energy at *m/z* 40–500 scan range. Metabolite identification was based on mass spectra of standard compounds and retention time or comparison with the in the National Institute of Standards and Technology (NIST). The unique ions from standard compounds were used for mass quantitation ions or qualifier ions.

### 3.3. Quantitation of Glucosinolate

Glucosinolates were analyzed following published methods [56]. Freeze-dried powdered samples from each of broccoli tissue (75 mg) were weighed into 2 mL screwcap vials (USA Scientific, Ocala, FL, USA) and mixed with 0.75 mL of 70% methanol. After heating at 95 °C for 10 min in a heating block, tubes were cooled on ice for 5 min, followed by the addition of 187.5 μL internal standard (1 mM glucosinalbin, isolated from *Sinapis alba*). Tubes were vortexed and centrifuged at 12,000*g* for 3 min at room temperature and the supernatants were collected. The pellets were extracted again with 0.75 mL of 70% methanol. The pooled extract was mixed with 0.15 mL of a mixture of 1 M lead acetate and 1 M barium acetate (1:1 *v/v*) and vortexed for protein precipitation. After centrifuging at 12,000*g* for 2 min, contents of each tube were poured into a drained Poly-Prep column containing DEAE Sephadex A-25 resin (GE Healthcare, Piscataway, NJ, USA) pre-charged with 1 M NaOH and 1 M pyridine acetate. After the sample solution passed through the resin, 3 mL of 0.02 M pyridine acetate was added followed by the addition of 3 mL of deionized (d^.^)H_2_O. Once the d^.^H_2_O passed through the resin, samples on the Poly-Prep column were incubated with 0.5 mL of sulfatase solution overnight at room temperature. Glucosinolates were eluted with 1.5 mL d^.^H_2_O. Tubes were vortexed and the eluent was filtered through a 0.22 μm nylon syringe filter into a 2 mL vial. A filtered sample of 50 μL was injected into a Nexera-i, LC 2040C ultra-high performance liquid chromatograph (UHPLC) (Shimadzu, Kyoto, Japan) equipped with a photodiode array detector. Glucosinolates were separated using a Kromasil RP-C18 column (250 mm × 4.6 mm) with mobile phase A (water) and B (100% acetonitrile) under following gradient conditions: 0 min 0% B, 7 min 4% B, 20 min 20% B, 35 min 25% B, 36 min 80% B, 40 min 80% B, 41 min 0% B, detected at 229 nm, and a flow rate of 1.5 mL/min. 

### 3.4. Quantitation of Myrosinase Activity and Nitrile Formation from Glucosinolate

Myrosinase activity and percentage of nitrile formation to total hydrolysis products from glucosinolate were measured to estimate ESP and ESM1 interaction based on published methods [57]. Myrosinase activity was estimated as the total amount of hydrolysis products produced within 60 min. One unit was defined as 1 μmol of total hydrolysis products release per min. Nitrile formation by samples was determined by incubating concentrated horseradish root extract with protein extracts of different broccoli samples. The horseradish extract was used as an exogenous substrate source of sinigrin and gluconasturtiin at a saturated level in order to minimize of the reaction with endogenous glucosinolate substrates from the different broccoli samples. Subsequently, hydrolysis products from sinigrin and gluconasturtiin were the dominant compounds detected from GC-MS. Horseradish extract was prepared by mixing 10 g of “1091” horseradish [56] with 100 mL of 70% methanol. This solution was centrifuged at 4000*g* for 5 min. The supernatant of was transferred to a beaker and boiled until all solvent was evaporated and reconstituted with 50 mL of deionized water. Freeze-dried sample powder (75 mg) was mixed with 1.5 mL of concentrated “1091” horseradish root extract [56] in 2 mL microcentrifuge tubes. After centrifugation at 12,000*g* for 2 min, 0.5 mL supernatant samples were transferred to 1.5 mL Teflon centrifuge tubes (Savillex Corporation, Eden Prairie, MN, USA) and 0.5 mL of dichloromethane was added. The tubes were placed upside down to minimize loss of volatile compounds at room temperature for 10 min. Then, tubes were vortexed and centrifuged at 12,000*g* for 4 min. The dichloromethane organic layer was injected into the GC-MS system described above to determine glucosinolate hydrolysis products. After an initial temperature hold at 40 °C for 2 min, the oven temperature was increased to 320 °C at 15 °C/min and held for 4 min. Injector and detector temperatures were set at 270 °C and 275 °C, respectively. The flow rate of the helium carrier gas was set at 1.2 mL/min. Standard curves of allyl isothiocyanate, 2-phenthyl isothiocyanate, and 3-phenylpropionitrile (Sigma-Aldrich, St Louis, MO, USA) were used for quantification. The standard curve from allyl isothiocyanate was also applied to quantify 1-cyano-2,3-epithiopropane. 

### 3.5. RNA Extraction and Quantitative Real-Time Polymerase Chain Reaction (qRT-PCR)

Total RNA was isolated from different broccoli samples using the RNeasy Mini Kit (Qiagen, Valencia, CA, USA) according to the manufacturer’s instructions. The quantity of RNA was measured using a NanoDrop 3300 spectrophotometer (Thermo Scientific). One microgram of the total RNA was reverse-transcribed with Superscript™ III First-Strand Synthesis SuperMix for qRT-PCR (Invitrogen, Carlsbad, CA, USA) according to the manufacturer’s instructions. The resulting cDNA samples were diluted to 1/10 their concentrations (*v/v*) for qRT-PCR. The list of the primers used can be found in Supplementary Table S1. The primers were synthesized by Integrated DNA Technologies (Coralville, IA, USA). Quantitative real-time PCR was carried out with the Power SYBR^®^ Green RT-PCR Master Mix (Qiagen) using a QuantStudio 3 (Applied Biosystems, Foster City, CA, USA) according to the manufacturer’s instructions. The relative expression ratio was determined with the Equation 2^−∆∆Ct^ using the *BoACT2* normalized ∆Ct values generated by the QuantStudio 3.

### 3.6. Carotenoid and Chlorophyll Analysis

Carotenoids and chlorophylls were extracted following methods outlined in our previous publication [57]. Freeze-dried powder (0.1 g) of each sample was extracted with 8.5 mL of acetone/methanol (2:1 *v/v*, containing 0.5% BHT). Then, 3 mL of hexane containing 0.5% BHT and internal standard (β-apo-8’-carotenal) was added followed by sonication in an ice bath for 20 min. After vigorous shaking, 8 mL of cold sodium chloride (1 M) was added, followed by centrifugation at 1800 rpm for 10 min for phase separation. The upper hexane layer was filtered through a 0.2 μm polytetrafluoroethylene (PTFE) syringe filter to an HPLC amber vial. UHPLC conditions for quantification were identical with those previously reported [57]. Each carotenoid and chlorophyll was identified using an authentic standard. For carotenoids, relative response factors based on the internal standard were used for quantification. An external standard curve was used to quantify chlorophylls. To estimate nutritional value related to provitamin A (β-carotene) of different tissues of broccoli, the RDA value of vitamin A was calculated on the basis of 100 g of fresh weight portion of broccoli with retinol activity equivalent conversion rate from dry weight using water content.

### 3.7. Mineral Analysis

Mineral concentrations were determined using published methods [58] with slight modification. Freeze-dried powder of each broccoli sample (0.5 g) was placed in a porcelain crucible and ashed at 550 °C overnight. Ashed samples were dissolved in 5 mL of 1 N nitric acid and samples were transferred to 25 mL volumetric flasks. All crucibles were rinsed with d^.^H_2_O, and the rinses were combined with the nitric acid digests. Each sample was adjusted to 25 mL with d^.^H_2_O. All flasks were shaken well, and the samples were filtered through filter paper (Grade 2, Fisher Scientific, Waltham, MA, USA) and stored at 4 °C until analysis. Mineral content was analyzed using inductively coupled plasma emission spectrometry (Optima 2100DV, Perkin Elmer Corp., Waltham, MA, USA). The plasma, auxiliary, and nebulizer argon gas flows were 12.0, 1.0, and 0.7 L/min, respectively, and the pump flow rate was 12 rpm. Mineral concentration in the sample was determined based on a standard curve of each element. Mineral concentration was calculated based on dry weight, and then converted to values based on fresh weight to calculate % RDA or adequate intake (AI). 

### 3.8. Analysis of Vitamins E and K

Vitamins E and K were extracted from 0.3 g of freeze-dried tissue following the method of Xiao et al. [59] with modifications. Each sample was mixed with 5 mL of d^.^H_2_O and 8 mL of isopropanol/hexane (3:2 *v/v*) in a 20 mL amber vial with a nitrogen stream for 5 s and extracted for 1 h in room temperature. Then, 10 μL of menaquinone (400 μg/mL) and 120 μL of α-tocopherol acetate (2 mg/mL) were added as internal standards. Samples were centrifuged at 1800 rpm for 10 min. The upper hexane layer was collected, and the samples were re-extracted with 5 mL of hexane as described above. The pooled hexane extract was completely dried under a nitrogen stream and reconstituted in 1 mL of hexane. Samples (0.8 mL) were loaded to 1.5 g of prepacked Florisil (6 mL glass tubes with PTFE frits, Restek Corp., Bellefonte, PA, USA) preconditioned with 5 mL of 30% ethyl ether in hexane followed by 8 mL of hexane, in order to separate vitamins E and K from other nonpolar organic compounds, such as chlorophylls and carotenoids. The columns were washed with 5 mL of hexane and then vitamins E and K were eluted with 10 mL of 30% ethyl ether in hexane. The solvent was completely dried under a nitrogen stream and the sample was reconstituted in 300 μL of hexane. All samples were filtered through a 0.2 μm PTFE syringe filter to a HPLC amber vial and kept at −20 °C until analysis. Vitamin E and K were quantified using UHPLC following published methods [57]. 

### 3.9. Total Phenolic Content and Antioxidant Capacity

Total phenolic content and antioxidant capacity were analyzed following the methods of Ku et al. [60] with minor modification. Freeze-dried samples (75 mg) were extracted in 6 mL of 70% methanol at room temperature for 10 min. After centrifuging, the supernatants were used for the total phenolic content and 2,2-diphenyl-1-picryl-hydrazyl-hydrate (DPPH) antioxidant capacity analysis. Vitamin C was used as a positive control (125 µg/mL) in DPPH assay. Results were expressed as a percentage of scavenging activity compared to control (extraction solvent). Samples were assayed in triplicate per biological replication. 

### 3.10. Statistical Analyses

All analyses were done using three replicates. Univariate analyses of variance (ANOVA) and least significant differences (LSD) were performed using JMP Pro 12 (SAS Institute, Cary, NC, USA) to evaluate differences in phytonutrient concentration between the different broccoli tissues. Heatmaps and biomarker selections were done by partial least squares discriminant analysis (PLS-DA) in MetaboAnalyst 3.5 [61].

## 4. Conclusions

We have undertaken a comprehensive phytonutrient assessment of broccoli by-products that will be useful for consumers and producers, as well as industries utilizing bioactive compounds. Although floret tissue of broccoli had the highest concentrations of glucoraphanin and most amino acids among the broccoli tissues evaluated, leaf tissue had higher essential nutrients, including β-carotene (provitamin A), vitamins E and K, the minerals Mn and Ca, as well as total phenolic content and DPPH antioxidant activity. Our results suggest that broccoli leaf tissue can be an excellent source for essential nutrients for foods. Further, evaluation of the metabolic pathways functioning to support the production and disposition of glucosinolates indicates that complex differences in pathway structure may underlie the observed patterns of glucosinolate concentrations in broccoli tissues. Expression patterns for florets, stems, and leaves each contained different elevated core genes, which may regulate the partitioning of glucosinolates among pools and, ultimately, influence the levels of glucosinolates and sulforaphanes in these tissues. These findings support future efforts to utilize broccoli agrowaste directly for human consumption as well as for sources of compounds for the nutraceutical and pharmaceutical industries.

## Figures and Tables

**Table 1:** Glucosinolate concentrations in different tissues of broccoli (μmol/g dry weight, DW).

Tissue	GIB	PRO	GRA	GNA	GER	GNS	GBC	4-OH-GBC	4-Methoxy-GBC	Neo-GBC	Total
Floret	2.26 ± 0.33a (6.52)	1.01 ± 0.11a (2.90)	11.25 ± 1.82a (32.45)	0.11 ± 0.02a (0.31)	0.08 ± 0.05b (0.23)	0.89 ± 0.29a (2.58)	1.54 ± 0.30a (4.45)	0.02 ± 0.01a (0.07)	0.58 ± 0.02a (1.66)	16.67 ± 1.64a (48.11)	34.66
Stem	0.97 ± 0.16b (13.04)	0.24 ± 0.04b (3.22)	3.79 ± 0.78b (50.88)	0.03 ± 0.00b (0.47)	0.89 ± 0.19a (11.97)	0.02 ± 0.00b (0.27)	0.10 ± 0.02b (1.40)	0.07 ± 0.02a (0.91)	0.16 ± 0.01b (2.17)	1.11 ± 0.47c (14.85)	7.45
Leaf	0.65 ± 0.10b (6.43)	0.02 ± 0.00c (0.18)	2.77 ± 0.59b (27.45)	0.04 ± 0.01b (0.36)	0.04 ± 0.01b (0.40)	0.11 ± 0.01b (1.08)	0.24 ± 0.05b (2.41)	0.26 ± 0.31a (2.56)	0.17 ± 0.03b (1.66)	5.78 ± 0.64b (57.35)	10.08

Data are expressed as mean ± SD (*n* = 3). Means were separated by least square difference (LSD) at *p* ≤ 0.05. Values in the parenthesis indicate the percentage (%) of each compound to the total glucosinolate concentration. Broccoli florets have 0.25 μmol/g DW of sinigrin and other tissues have lower than 0.1 μmol/g DW. This is included only in the total glucosinolate concentration. GIB: glucoiberin; PRO: progoitrin; GRA: glucoraphanin; GNA: gluconapin; GER: glucoerucin; GNS: gluconasturtiin; GBC: glucobrassicin.

**Table 2:** Carotenoid and chlorophyll concentrations in the different broccoli tissues (μg/g DW).

Tissue	Carotenoids					Chlorophylls		
	β-carotene	Violaxanthin	Neoxanthin	Lutein	Total	Chlorophyll *a*	Chlorophyll *b*	Total
Floret	30.6 ± 4.6b (16.9)	34.7 ± 2.4b (19.1)	30.2 ± 1.3b (16.7)	85.5 ± 8.3b (47.2)	181.0	852.1 ± 105.5b (86.4)	134.6 ± 14.3b (13.6)	986.7
Stem	0.0c	0.0c	4.8 ± 8.3c (30.8)	10.8 ± 9.5c (69.2)	15.6	143.7 ± 51.6c (86.6)	22.2 ± 9.2c (13.4)	165.8
Leaf	248.4 ± 28.9a (22.7)	206.3 ± 20.3a (18.8)	156.2 ± 10.6a (14.3)	484.1 ± 33.2a (44.2)	1095.0	4477.9 ± 408.6a (85.2)	780.9 ± 56.3a (14.8)	5258.8

Different letters indicate significant differences among tissues within the column by LSD *p* ≤ 0.05. Numbers in the bracket indicate the percentage (%) of individual carotenoid/chlorophylls to total carotenoids or chlorophylls.

**Table 3:** Essential mineral element concentrations in different tissues of broccoli.

Tissue	Fe	Zn	Mn	Cu	Ca	Mg	P	Na	K
	μg/g DW				mg/g DW				
Floret	45.83 ± 0.83a (3.5)	54.00 ± 3.01a (6.8)	18.83 ± 0.17b (11.4)	0.29 ± 0.08a (0.45)	4.65 ± 0.10b (5.4)	1.78 ± 0.03a (5.9)	7.01 ± 0.12a (13.9)	0.39 ± 0.03ab (0.4)	145 ± 22a (3.1)
Stem	15.83 ± 3.08b (0.9)	22.67 ± 5.17b (2.2)	7.00 ± 0.29c (3.3)	0.24 ± 0.12a (0.29)	7.10 ± 0.36b (5.3)	1.67 ± 0.19a (4.3)	5.07 ± 0.18b (7.7)	6.43 ± 0.16a (0.5)	182 ± 7a (3.9)
Leaf	40.50 ± 1.26a (4.3)	23.33 ± 2.42b (4.1)	26.17 ± 1.69a (22.0)	0.21 ± 0.11a (0.46)	28.99 ± 1.97a (46.6)	1.33 ± 0.13a (6.1)	3.42 ± 0.08c (9.4)	2.63 ± 0.03b (0.3)	136 ± 72a (2.9)

Different letters indicate a significant difference among tissues within the same column by LSD at *p* ≤ 0.05. Numbers in the bracket indicate the percentage (%) of RDA of each mineral based on 100 g of fresh weight.

## Supplementary Materials

Supplementary materials are available online http://www.mdpi.com/1420-3049/23/4/900/s1.
