# Supplementary material for: Comparative Phytonutrient Analysis of Broccoli By-Products: The Potentials for Broccoli By-Product Utilization
Source: Molecules. 2018 Apr 13;23(4):900. doi: 10.3390/molecules23040900 (PMC6017511; doi:10.3390/molecules23040900)

Supplementary Table 1. Broccoli glucosinolate biosynthesis and myrosinase mediated hydrolysis related gene primer list.

Gene name	Primer name[a]	Sequence
Actin 2	Bol030974 F	TCCCGAGAGGAAGTACAGTGTCT
	Bol030974 R	GAGATCCACATCTGCTGGAATG
FMO GS-OX2	Bol010933v2 F	CCGGAGCATCTGGATTAATAGC
	Bol010933v2 R	CACTTGTTTCTCCCGCTCAAA
FMO GS-OX5.1	Bol031353v2 F	GAGCTTTGACTCCGCTATGGA
	Bol031353v2 R	TTTACTTGTAGCGCACGTTTCG
AOP2	AY044425.1 F	TGGGTGCAGACACTCCTCAA
	AY044425.1 R	CCCACTTCTCACTTCCTGGTTT
GS-OH	Bol033373 F	GCTTGTTGATGCTCTGTCATTGT
	Bol033373 R	TGGCGCCGAGCGTTAG
CYP81F1	Bol028913 F	CCGAGACATTCCGGCTATTC
	Bol028913 R	CATGTCCTCCGTCGGTCTTC
CYP81F4	Bol032712v2 F	TCCCTCTCCGCCTCACTCT
	Bol032712v2 R	GGTGGACGGGAGGTTTAATGA
IGMT1	Bol007030 F	GGACCGGATGCTTCGTCTAC
	Bol007030 R	TCTCTCGCCCTTTCCAAACTT
TGG1	Bol017328v2 F	GTGCCTACGAGAGGCTATTCAAC
	Bol017328v2 R	GCCGTAACATCTTTCATCAACCT
TGG2	Bol028319v2 F	CGAACTCAACGCTACTGGTTACA
	Bol028319v2 R	TACTCCCCTGCTCCTCTTTCC
ESP1	Bol006378 F	CTACACGACTGCTACCGTCTATGG
	Bol006378 R	GGTTGTTGGTGGGACGTTTT
ESP2	Bol039072v2 F	TGTTTGGACATGCGGTTGTG
	Bol039072v2 R	CGTCCCTGGTCCCAAATG
ESM1	Bol005067v2 F	TCCGATGTTGAACCAGTTTGC
	Bol005067v2 R	CGAAGGATGGCGTTGTAGAAA
MYB34.2	Bol007760 F	GCTCAAACCGGTGGCAAA
	Bol007760 R	CGTCAAGATCATCGGAGAAAGA
MYB122	Bol026204 F	CTTCCCGACAAAGCTGGACT
	Bol026204 R	TTGGCTAAACTCACCACGCT
SOT16	Bol039395 F	TTCGACGACGCCACGAA
	Bol039395 R	CTCCACGTAAGGCACGAACTC
UGT74B1	Bol005786 F	CGACGGCCACGACTTCAT
	Bol005786 R	GCTTGAAGGATTCGGAGTATGC
CYP79B2	Bol032767 F	GATGAAATTAAACCCACCATTAAGGA
	Bol032767 R	GCCATGGCCCATTCGA

Supplementary Figure 1. Broccoli ('Gypsy' cultivar, grown in field) individual tissue biomass (Fresh weight) percentage to total biomass. The data collected from 7 individual mature broccoli plants. Average of total biomass was 776 g per plant.

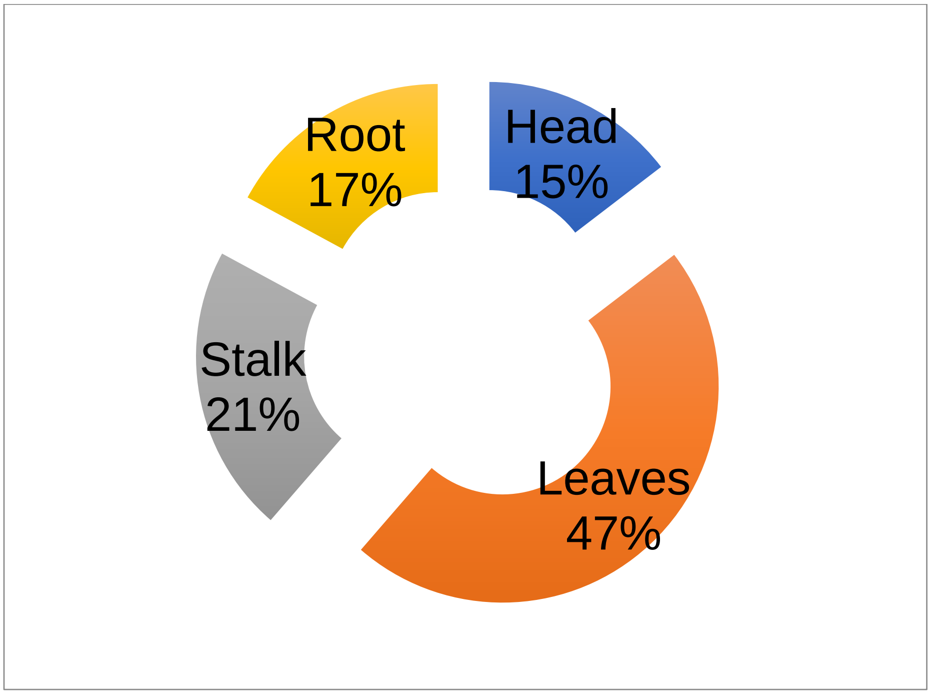

Supplementary Figure 2. Total phenolic content gallic acid equivalent (GAE) mg per g of DW (A) and DPPH antioxidant activity (B) in different tissues of broccoli (12.5 mg DW/mL). Vitamin C was used as positive control (125 μg/mL).

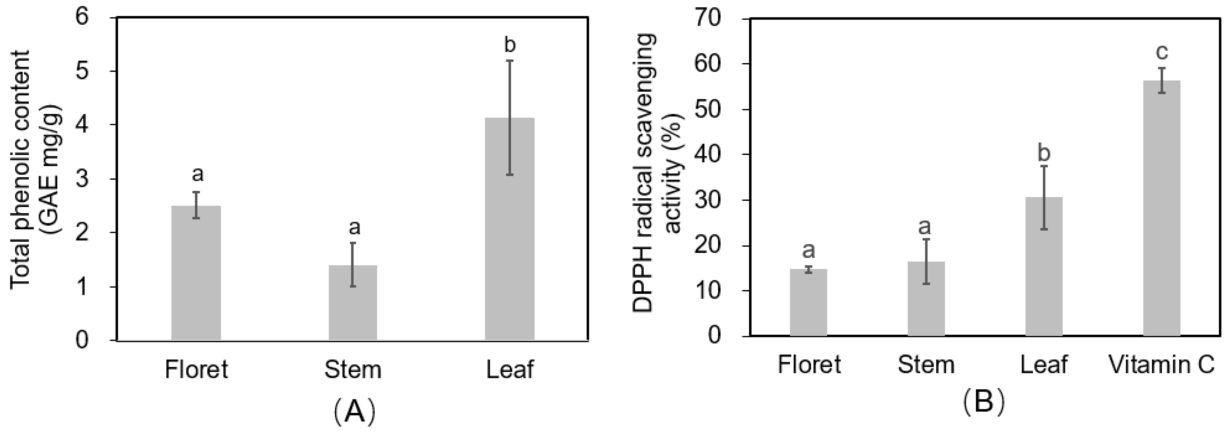

Supplement: Supplementary file 1 [file molecules-23-00900-s001.pdf]
